# MIL-CT: Multiple Instance Learning via a Cross-Scale Transformer for Enhanced Arterial Light Reflex Detection

**DOI:** 10.3390/bioengineering10080971

**Published:** 2023-08-16

**Authors:** Yuan Gao, Chenbin Ma, Lishuang Guo, Xuxiang Zhang, Xunming Ji

**Affiliations:** 1 Department of Biomedical Engineering, School of Biological Science and Medical Engineering, Beihang University, Beijing 100191, China; 2 Department of Ophthalmology, Xuanwu Hospital, Capital Medical University, Beijing 100053, China; 3 Shen Yuan Honors College, Beihang University, Beijing 100191, China; 4 Department of Ophthalmology, Beijing Tiantan Hospital, Capital Medical University, Beijing 100050, China

**Keywords:** deep learning, multiple instance learning, cross-scale transformer, arteriosclerosis, fundus image

## Abstract

One of the early manifestations of systemic atherosclerosis, which leads to blood circulation issues, is the enhanced arterial light reflex (EALR). Fundus images are commonly used for regular screening purposes to intervene and assess the severity of systemic atherosclerosis in a timely manner. However, there is a lack of automated methods that can meet the demands of large-scale population screening. Therefore, this study introduces a novel cross-scale transformer-based multi-instance learning method, named MIL-CT, for the detection of early arterial lesions (e.g., EALR) in fundus images. MIL-CT utilizes the cross-scale vision transformer to extract retinal features in a multi-granularity perceptual domain. It incorporates a multi-head cross-scale attention fusion module to enhance global perceptual capability and feature representation. By integrating information from different scales and minimizing information loss, the method significantly improves the performance of the EALR detection task. Furthermore, a multi-instance learning module is implemented to enable the model to better comprehend local details and features in fundus images, facilitating the classification of patch tokens related to retinal lesions. To effectively learn the features associated with retinal lesions, we utilize weights pre-trained on a large fundus image Kaggle dataset. Our validation and comparison experiments conducted on our collected EALR dataset demonstrate the effectiveness of the MIL-CT method in reducing generalization errors while maintaining efficient attention to retinal vascular details. Moreover, the method surpasses existing models in EALR detection, achieving an accuracy, precision, sensitivity, specificity, and F1 score of 97.62%, 97.63%, 97.05%, 96.48%, and 97.62%, respectively. These results exhibit the significant enhancement in diagnostic accuracy of fundus images brought about by the MIL-CT method. Thus, it holds potential for various applications, particularly in the early screening of cardiovascular diseases such as hypertension and atherosclerosis.

## 1. Introduction

In recent years, atherosclerosis has become a widespread vascular lesion worldwide. According to a recent analysis, approximately 50.20% of people aged 30–79 will be affected by atherosclerosis in 2020, which is equivalent to around 1940.25 million people [1]. The prevalence of arterial dysfunction is projected to continue rising as the population ages. It is estimated that by 2050 there will be a total of 128 million individuals aged 80 or older [2]. Early systemic atherosclerosis often does not exhibit obvious clinical symptoms, but as the disease progresses, it can cause impaired blood circulation or even lead to infarction. Retinal vessels can reflect abnormalities in the systemic vascular system, and enhanced retinal arterial light reflex (EALR) is an early manifestation of systemic atherosclerosis [3]. Moreover, the retinal vasculature is one of the few vessels that can be observed noninvasively [3]; thus, regular testing of EALR can aid in understanding the degree of systemic atherosclerosis [4] and facilitate timely medical intervention to prevent further deterioration.

Ophthalmologists commonly use 2D color fundus images and 3D optical coherence tomography images for screening and diagnosing conditions [5]. However, compared to optical coherence tomography, fundus images are more suitable for large-scale screening due to their noninvasive and painless nature. Fundus images are obtained by photographing the inner wall of the eye from different angles using a fundus camera. These images provide direct visualization of retinal arteriovenous vasculopathy and other features of fundus disease [5]. Hence, ophthalmologists can determine the presence of retinopathy by examining fundus images [5]. EALR is characterized by alterations in the diameter and brightness of the retinal arterioles [3], resulting in increased optical density of the arterial wall and narrowing of the arteriole lumen, leading to enhanced reflection. This phenomenon is observed in different stages as changes in the retinal arterial reflection band and reflection brightness [3,4]. When EALR occurs, the arterial reflective band widens and the color changes from normal red to a metallic bright copper shade [4]. With further progression of atherosclerosis, the vessels will acquire a white silvery reflective appearance [4].

Currently, the diagnosis of EALR relies on empirical observation of fundus images to determine the presence of EALR by comparing the reflection degree and width of arterial reflective bands with those of blood vessels. However, this traditional diagnostic method requires doctors to spend a significant amount of time analyzing fundus images, resulting in high workload, low efficiency, and impracticality for large-scale ophthalmic disease screening. Consequently, computer-aided diagnosis technology based on deep learning has emerged as an effective solution for fast, fully automated, and highly accurate EALR detection [6,7,8,9]. Deep learning networks have the ability to develop more abstract high-level features, automatically capturing the most crucial and distinctive data characteristics in the images, thereby improving the accuracy of EALR detection. Nevertheless, existing studies typically employ a two-stage process: the design of deep learning networks to segment retinal vessels at the pixel level, followed by the identification of arteriovenous vessels and the extraction of morphological parameters such as color and brightness. Finally, classifiers are employed to identify lesion symptoms like EALR. These methods increase the complexity of algorithms, and the accuracy of EALR detection heavily relies on the accuracy of the vessel segmentation stage. Specifically, previous research methods, including the Gabor-based approach and the deep learning network-based retinal vessel segmentation method introduced by Henry et al., have shown limitations in accurately segmenting retinal vessel edges [7]. Similarly, the method proposed by Fu et al., which combines a deep learning network with a fully connected conditional random field, has struggled to differentiate arteriovenous vessels [8]. Additionally, Yan et al.’s recent method, which utilizes segmental level loss, demonstrated reduced segmentation accuracy in cases where vessels were obstructed by diseased tissue [9].

The contributions of MIL-CT are as follows: Firstly, as the earliest work on fundus image-based EALR detection, our proposed MIL-CT constructs a transformer model using a multi-head cross-scale attention (MHCA) fusion mechanism to enhance the multi-scale global modeling capability of the transformer, thereby improving classification performance. Secondly, by leveraging the multi-level hybrid architecture of the transformer, our approach (MIL-CT) can extract features at different scales, achieving higher classification accuracy by utilizing varying granularity in features and integrated distribution patterns. Finally, by introducing a multi-instance learning (MIL) head to leverage the features extracted from individual patch tokens, we can easily integrate them into the MIL-CT model, further enhancing classification accuracy.

To train the proposed MIL-CT model, we performed pre-training using a large-scale fundus dataset, which significantly improved the performance of the model in the downstream EALR detection task. Evaluating our method on the collected EALR dataset, we demonstrated superior performance and reliable interpretability compared to existing state-of-the-art (SOTA) methods. This innovation also provides valuable insights for the early diagnosis of cardiovascular diseases, such as atherosclerosis, as retinal vessels exhibit unique spatial variability and global correlation.

## 2. Methodology

### 2.1. Overall Framework

**Figure 1:**
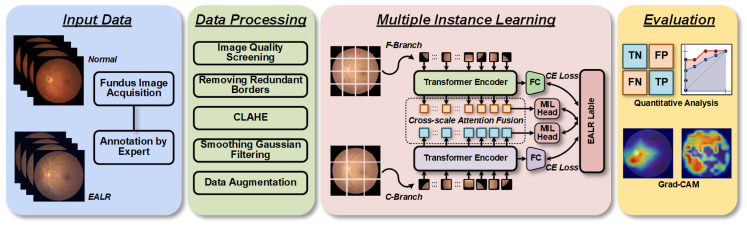
Workflow diagram of the proposed EALR detection framework.

Figure 1 depicts the overall framework of the MIL-CT approach. Initially, labeled fundus images are acquired and utilized as raw data for training and validation purposes. Next, these images undergo preprocessing to ensure compatibility with the input of the model. The processed data are then fed into the MIL-CT model, which leverages an MIL strategy. Specifically, the multi-scale feature extractor of the MIL-CT model is based on the transformer, enabling effective interaction among input sequences of different scales. To enhance the utilization of patch tokens from the extracted multi-scale images and capture complementary information at specific scales, we introduce a novel plug-and-play MIL module. Ultimately, the effectiveness of our proposed approach is validated through quantitative evaluation and interpretability analysis.

### 2.2. Cross-Scale Transformer

In traditional convolutional neural networks (CNNs), feature extraction is accomplished by stacking convolutional and pooling layers. This results in feature maps with diminishing scale but increasing channel dimensions. Typically, feature maps closer to the input exhibit larger scale, lower channel dimensionality, and contain more detailed information. On the other hand, feature maps farther from the input have smaller scale, larger dimensionality, and contain more semantic information. In the context of detecting EALR in fundus images, the fusion of multi-scale features proves advantageous for performance enhancement. However, the vanilla vision transformer (ViT) fails to efficiently utilize such multi-scale features [10]. Therefore, we propose a transformer model that incorporates cross-scale feature fusion to facilitate effective interaction between input sequences at different scales.

#### 2.2.1. Vanilla ViT

**Figure 2:**
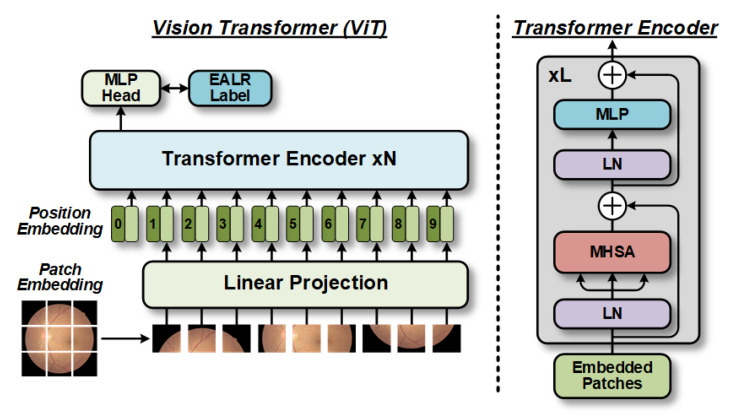
Vanilla ViT divides the input fundus image into multiple patches. Each patch is then projected into a fixed-length vector and fed into the transformer encoder. To perform classification on the images, a dedicated classification token is included in the input flattened patches. The output corresponding to this token represents the final ELAR prediction.

Vanilla ViT [10] is a scientific approach that can be employed for feature extraction in EALR detection using fundus images as a backbone, as shown in Figure 2. The method begins by partitioning the image into fixed-sized patches. These patches are then transformed into sequences of token patches through linear projection. To preserve crucial location information relevant to vision applications, position embedding is incorporated into each token, including classification tokens. All tokens undergo processing through a sequence of transformer encoder blocks and are ultimately classified using the classification tokens. Each transformer encoder block consists of a multi-headed self-attentive (*MHSA*) mechanism and a feedforward network (*FFN*). The FFN comprises two linear layers, with a hidden layer dimensionality expansion rate denoted as *e*. Following the first linear layer, a GELU nonlinear activation function is utilized. Layer normalization (*LN*) is applied before and after each block, which are connected by residual shortcuts.

To ensure compatibility between the fundus images and transformer encoders, we undertook preprocessing of the input data. We use $X={\left\{x_{i}\right\}}_{i=1}^{n}\in {\mathbb{R}}^{w\times h\times r}$ to represent the fundus images in the database (where *w*, *h*, and *r* denote the dimensions of image width, height, and color channels, respectively), and their corresponding labels are represented by $Y\in {\mathbb{R}}^{n\times 1}$ (i.e., indicating whether they are EALR). Each fundus image was divided into patches of size *p* × *p*, with *p* set to 16 in this study based on the optimal experimental setup [10]. Consequently, *n* = (w × *h*)/(*p*^2^) patches were obtained as input from a single image. This allows us to express the input for the transformer as *x*_0_, and the processing of the *i*-th block can be represented as follows:

(1) $$x_{0}=\{x_{cls}||x_{patch}\}+x_{pos}$$

(2) $$y_{i}=x_{i-1}+MSHA\left(LN(x_{i-1})\right)$$

(3) $$x_{i}=y_{i}+FFN\left(LN(y_{i})\right)$$

where $x_{cls}\in {\mathbb{R}}^{1\times q}$ and $x_{patch}\in {\mathbb{R}}^{n\times q}$ are classification and patch tokens, respectively, $x_{pos}\in {\mathbb{R}}^{(n+1)\times q}$ is the position embedding with *q* as the dimension, and {⋅||⋅} is the concatenate operation. In particular, the mathematical representation of *LN* is

(4) $$LN(x)=\frac{x-E(x)}{Var(x)+\varepsilon }\cdot \gamma +\beta$$

where *γ* and *β* are the learnable parameters of the *LN* operation.

ViTs stand out from traditional CNNs due to their unique design feature: the inclusion of classification tokens. Unlike CNNs, which usually obtain final feature embedding by averaging features across spatial locations, ViTs incorporate classification tokens that interact with patch tokens in each transformer encoder, thus serving as the ultimate embedding representation. As a result, the classification tokens are seen as a way to merge all patch token information [11]. This distinct architectural design enables ViTs to achieve cross-scale information fusion for both branches.

#### 2.2.2. Multi-Scale Feature Extraction

**Figure 3:**
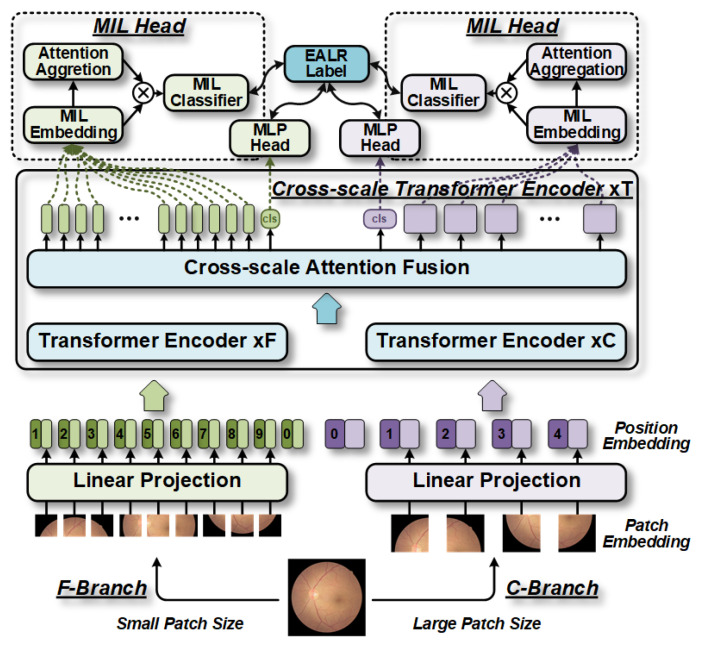
The architecture of the proposed CT model. The model divides the fundus image into patches of various sizes simultaneously. These patches are then fed into two branches with similar structures: the C-Branch and F-Branch. Generally, the F-Branch is shallower than the C-Branch to promote smoother learning and balance computational costs. The MHCA fusion module efficiently facilitates interaction between the feature vectors of the classification tokens and patch tokens from the two branches, allowing the model to capture information in the multi-granularity perceptual domain.

The choice of patch size in a vanilla ViT has a significant impact on model accuracy and complexity. It is generally believed that using finer-grained perceptual patches can lead to better performance at the cost of increased floating-point operations per second (FLOPs) and memory consumption. For instance, studies have shown that a vanilla ViT with a patch size of 16 achieves a 6% improvement compared to a coarser-grained ViT with a patch size of 32, though it requires four times more FLOPs [11]. Motivated by this, our proposed method aims to leverage multi-grain patch size perception to extract multi-scale features from fundus images and design different feature extractor sizes to balance complexity between branches. As illustrated in Figure 3, we introduce multi-grain size perception, or embedded patch size, into a multi-branch ViT to obtain the cross-scale features of fundus images. We then devise a cross-scale attention mechanism to fuse the extracted information effectively from each branch.

Specifically, we design a cross-scale feature extractor that comprises multiple transformer encoders at different scales. Each encoder consists of two branches: a coarse-grained main branch (C-Branch) and a fine-grained complementary branch (F-Branch). The C-Branch employs a larger patch size, uses more transformer encoders with wider embedding dimensions, and processes coarse-grained retinal information. In contrast, the F-Branch uses a smaller patch size, has fewer encoders, and uses smaller embedding dimensions to process fine-grained retinal information. These two branches employ multi-head cross-scale attention (MHCA) fusion to perform EALR detection using features extracted from different scales. To capture the location information between patches in the fundus image, we adopt an approach similar to the vanilla ViT [10] and introduce learnable position embeddings for each token of each branch prior to the cross-scale transformer (CT) encoder.

#### 2.2.3. Cross-Scale Attention Fusion

The cross-scale attention (CA) mechanism in our proposed methodology enhances the fusion of multi-scale features by efficiently combining classification tokens and patch tokens from different branches. This mechanism builds upon the conventional MHSA approach, which calculates the similarity between patch tokens to adjust input information. To extract retinal information from various perceptual domains, we introduce branch-specific classification tokens as agents within the CA mechanism. These tokens facilitate fusion by exchanging information with patch tokens from other branches. Since the classification token has already learned abstract information from all the patch tokens within its own branch, interacting with a patch token from another branch enables the smooth flow of information between different branches.

Once the patch tokens from other branches are fused with the classification token, they are projected back to their respective branches. This enriches the representation of each patch token, capturing valuable cross-scale information. Subsequently, at the next transformer encoder, the classification token interacts with its own patch tokens once again. This iterative process further promotes the transfer of knowledge learned from other branches, enhancing the representation of each patch token with comprehensive information. By employing the CA mechanism, our methodology effectively integrates information across different scales, enabling enhanced feature representation and better diagnostic accuracy in detecting early arterial lesions.

**Figure 4:**
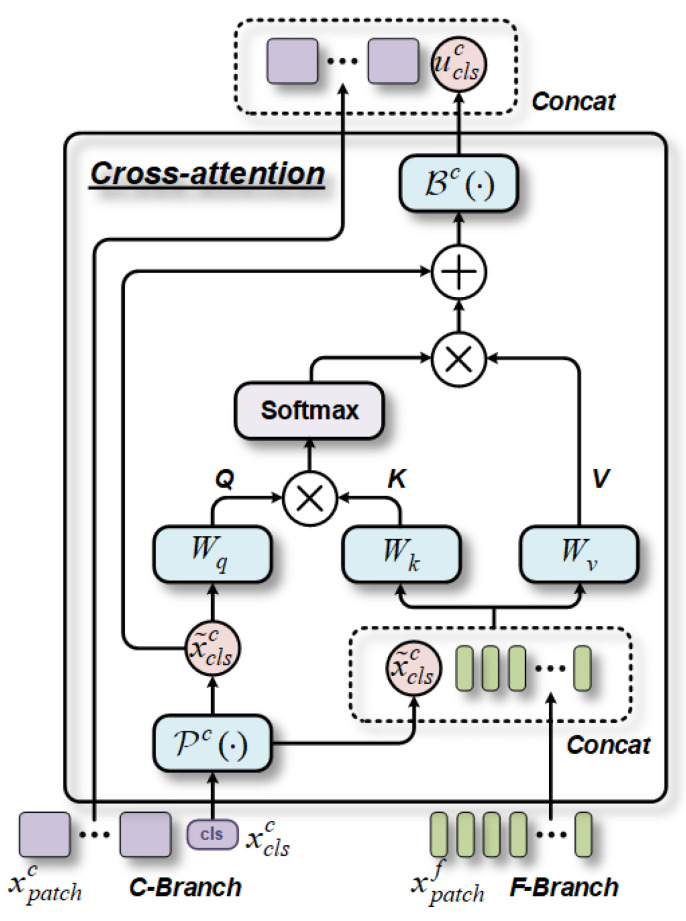
Schematic diagram of the CA fusion module in the C-Branch. In this module, the classification tokens in the C-Branch serve as query tokens in the self-attentive mechanism and effectively interact through similarity weight assignment. Similarly, the F-Branch undergoes a similar mirroring process.

Next, we describe the CA module for the C-Branch, as depicted in Figure 4, and replicate the same process for the F-Branch by simply exchanging the indexes “*f*” and “*c*”. By doing so, we can apply similar attention mechanisms at different scales to achieve the fusion of multi-scale features. Initially, the classification tokens of the C-Branch are fused with the patch tokens of the F-Branch through a concatenate operation:

(5) $${\hat{x}}^{c}=\{P^{c}(x_{cls}^{c})||x_{patch}^{f}\}$$

where $P^{c}(\cdot )$ is the projection function for dimension alignment. Then, *CA* can be performed between ${\hat{x}}^{c}$ and $x_{cls}^{c}$ as

(6) $$CA({\hat{x}}^{c},x_{cls}^{c})=softmax(QK^{T}/\sqrt{q/t})\cdot V$$

where the softmax function is used to normalize the similarity score, *t* is the number of heads, and query *Q*, key *K*, and value *V* can be expressed as

(7) $$\left\{\begin{array}{l} Q=P(x_{cls}^{c})\cdot W_{q}\\ K={\hat{x}}^{c}\cdot W_{k}\\ V={\hat{x}}^{c}\cdot W_{v} \end{array}\right.$$

where $W_{q},W_{k},W_{v}\in {\mathbb{R}}^{(c/t)\times c}$ are learnable parameters. Note that by utilizing only classification tokens in the query, the computational and memory complexity of generating the attention map in *CA* becomes linear rather than quadratic, as seen in the MHSA mechanism. This modification enhances the overall efficiency of the process. Furthermore, we have substituted the FFN in the vanilla ViT with a structure that incorporates LN and residual shortcuts. As a result, the final output *z^c^* can be represented as follows:

(8) $$z^{c}=\{B^{c}(u_{cls}^{c})||x_{patch}^{c}\}$$

where $B^{c}(\cdot )$ is the back-projection function for dimension alignment. We also obtain the united attention score $u_{cls}^{c}$ from the *MHCA* module:

(9) $$u_{cls}^{c}=P^{c}(x_{cls}^{c})+MHSA\left(LN\left(\left\{P^{c}(x_{cls}^{c})||x_{patch}^{f}\right\}\right)\right)$$

Similarly, the process of merging the classification token from the F-Branch with the patch token from the C-Branch is identical to the aforementioned process, thereby facilitating efficient interaction of cross-scale information. It is important to note that the proposed MHCA fusion mechanism in our MIL-CT method shares similarities with CrossViT [11] in terms of using a multi-head attention mechanism. However, there are key differences in how we utilize this mechanism. As shown in Figure 3 of the revised manuscript, we primarily employ the CT module in the MIL-CT to extract multi-scale visual features from fundus images. The CT module ensures minimal information loss while providing the MIL module with patch tokens of the different granularity perceptual domains required for instance embedding. Additionally, the MHCA fusion mechanism in MIL-CT is fixed as a single-layer MHCA, as we found that utilizing a single layer of cross-attention was more effective for our specific task.

### 2.3. Multiple Instance Learning

As previously mentioned, our study utilizes a CT architecture to facilitate the fusion of multi-scale features. This is achieved through effective interaction between classification tokens from one branch and patch tokens from another branch. However, it is also important to refine the information of patch tokens within a single feature extraction branch. This is especially crucial when considering that retinal diseases may manifest in various locations [6], leading to varying contributions from different patches.

**Figure 5:**
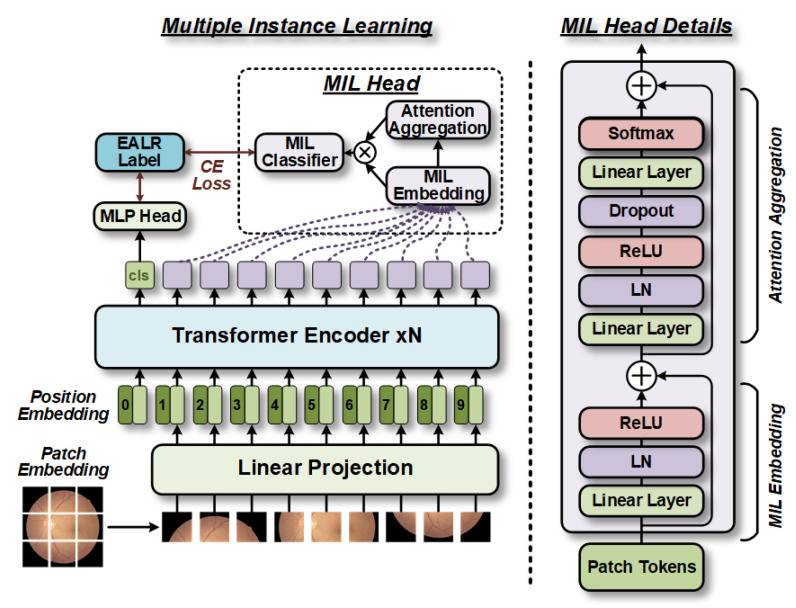
Schematic diagram of the proposed plug-and-play MIL module. In this module, the patch tokens are treated as instance relations. After low-dimensional embedding, an attention aggregation function is used to assign weights to the instance embeddings. The aggregated bag representations are then fed into a linear classifier to obtain the final bag-level probabilities. This approach provides complementary information to the predicted probabilities generated by the classification tokens.

To address this issue, we have adapted the bag—instance relationships presented in multiple instance learning (MIL) [12,13] to the image—patch relationships in ViTs. We have introduced the MIL approach into the CT structure to leverage the information shared among all patch tokens within each specific perceptual domain branch. Consequently, as shown in Figure 5, the MIL scheme involves the following three main steps: (1) construction of low-dimensional embeddings for all patch tokens within a given branch; (2) obtainment of bag representations through aggregation functions; and (3) determination of the final probabilities at the bag level using bag-level classifiers.

Similarly, we will illustrate the data flow chart of MIL by using the C-Branch as an example, while applying the same operation to the F-Branch.

#### 2.3.1. Low-Dimensional Embedding

Low-dimensional embedding learns meaningful representations of patch tokens and extracts the features most relevant to retinal pathology, thus reducing redundant information. Assuming that the bag—instance relationship containing coarse-grained perceptual domain information output in the C-Branch is denoted as $Z^{c}=\{z_{1}^{c},z_{2}^{c},\ldots ,z_{n}^{c}\}$, then for each obtained feature vector $z_{i}^{c}\in {\mathbb{R}}^{d}$ it will be fed into a low-dimensional embedding of dimension *m*, which consists of a linear layer, *LN*, and ReLU activation function:

(10) $$h_{i}^{c}=\max\left(LN(W_{linear}^{T}z_{i}^{c}),0\right)$$

where $W_{linear}\in {\mathbb{R}}^{d\times m}$ represents the weight of the linear layer.

#### 2.3.2. Attention Aggregation Function

Due to the varying contributions of different patch tokens to the bag, we aim to maximize their utilization while effectively distinguishing their dissimilarities. To achieve this, as illustrated in Figure 5, we propose an attention module that incorporates a bilinear layer to derive the spatial weight matrix of the instance embedding. The detailed procedure is described below:

(11) $$\alpha_{i}^{c}=softmax\left(W_{l2}^{T}\max\left(LN(h_{i}^{c}W_{l1}^{T}),0\right)\right)$$

where $W_{l1}\in {\mathbb{R}}^{l\times m}$ and $W_{l2}\in {\mathbb{R}}^{l\times 1}$ represent the weight of the two linear layers. The attention weights can be assigned to instance embeddings, thereby highlighting the distinct contributions made by different instances. The aggregated bag representation $A^{c}$ is defined as

(12) $$A^{c}=\sum_{i=1}^{n}\alpha_{i}^{c}h_{i}^{c}$$

#### 2.3.3. Bag-Level Classifier

A linear bag-level classifier is used to estimate the final bag-level probability $P^{c}$, mathematically represented as

(13) $$P^{c}=W_{bag}^{c}A^{c}$$

where $W_{bag}^{c}\in {\mathbb{R}}^{2\times l}$ represents the weight of the bag-level classifier.

By following the same procedure, we can easily derive the final bag-level probability $P^{f}$ for the F-Branch. These probabilities can be seamlessly combined to yield estimates of the probabilities for the MIL head:

(14) $$P^{mil}=\{P^{c}||P^{f}\}$$

### 2.4. Pre-Training and Fine Tuning

In order to maximize the utilization of the generalization bias learned from the fundus image dataset and expedite the model training process, our study employed a large fundus image classification dataset, specifically Kaggle [14], to pre-train the proposed MIL-CT model. Subsequently, the pre-trained MIL-CT model was fine-tuned for the downstream task of EALR detection using our dataset.

Throughout the training process, we formulated a jointly optimized objective loss function to guide the learning process of specific branches in the model:

(15) $$L(y^{gt},y^{mlp},y^{mil})=-\eta \sum_{j=1}^{2}y_{j}^{gt}log(y_{j}^{mlp})-(1-\eta )\sum_{j=1}^{2}y_{j}^{gt}log(y_{j}^{mil})$$

where *y^gt^* represents the ground-truth label and *η* is the loss hyperparameter. Meanwhile, *y^mlp^* and *y^mil^* represent the estimations made by the multi-layer perceptron (MLP) classifier and the MIL bag-level classifier, respectively. Specifically, for each scale branch, we extract information from the patch tokens and classification tokens based on the aforementioned cross-entropy-based joint optimization loss function. This loss function assigns weights to both the MIL classifier and the MLP header using the hyperparameter *η*. The losses obtained from each scale branch are then summed to derive the overall objective optimization function.

## 3. Experimental Setup

### 3.1. Data Description

We conducted a study on the detection of EALR in fundus images. For this purpose, we utilized two datasets: the publicly available pre-trained Kaggle dataset [14] and the collected EALR dataset. The Kaggle dataset was obtained from the 2015 Diabetic Retinopathy Detection Competition, organized by Kaggle and EyePACS [14]. The combined dataset comprises a total of 86,729 fundus image samples, with 33,133 training samples and 53,596 test samples. These images are classified into five levels based on the severity of diabetic retinopathy and have varying resolutions of approximately 3500 × 2500 pixels. In addition to these datasets, we also created a new dataset called EALR, which consists of 1579 fundus images taken from 1114 patients at the Xuanwu Hospital Ophthalmology Department between January 2014 and June 2022. Each fundus image in the EALR dataset underwent review and labeling by two ophthalmologists and a retinal specialist.

**Figure 6:**
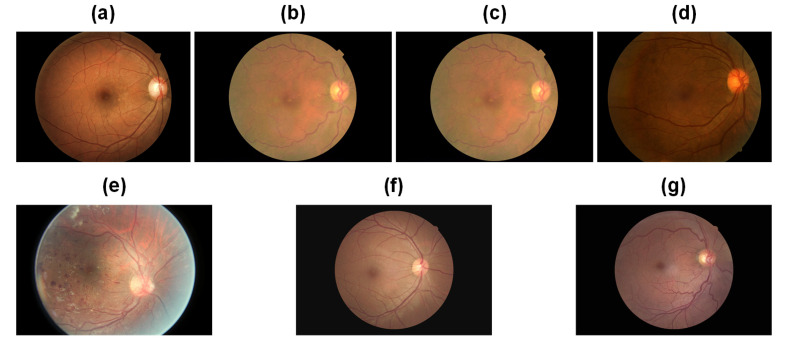
The five categories in the Kaggle dataset are (**a**) normal, (**b**) mild NPDR, (**c**) moderate NPDR, (**d**) severe NPDR, and (**e**) PDR. The two categories in the EALR dataset are (**f**) normal and (**g**) EALR.

Table 1 presents comprehensive statistical information regarding the datasets. The Kaggle dataset categorizes fundus images into five classes, with values ranging from 0 to 4 denoting normal, mild non-proliferative DR (NPDR), moderate NPDR, severe NPDR, and proliferative diabetic retinopathy (PDR), respectively. The corresponding samples are depicted in Figure 6, specifically Figure 6a–e. In contrast, the EALR dataset classifies fundus images into two stages, where 0 represents normal and 1 indicates the detection of EALR. These samples can be observed in Figure 6, specifically Figure 6f,g. It is worth noting that both datasets exhibit inter-class imbalance, a commonly encountered challenge in medical image datasets. Specifically, class 0 (lesion-free) samples account for 72.61% and 64.53% of the total number of samples in the Kaggle dataset and the EALR dataset, respectively. On the other hand, class 4 and EALR samples make up only 2.21% and 35.47% of the total samples, respectively. This data distribution significantly deviates from that of natural image datasets. The imbalance in data distribution poses a substantial problem during the training process of traditional deep learning models. Such models tend to predict categories with higher frequency, which can lead to misdiagnosis and delayed treatment, potentially worsen patient outcomes, or result in more serious consequences, especially in medical image classification. Therefore, addressing the data imbalance issue is a crucial aspect that requires our utmost attention in this research.

### 3.2. Data Processing

#### 3.2.1. Image Quality Screening

**Figure 7:**
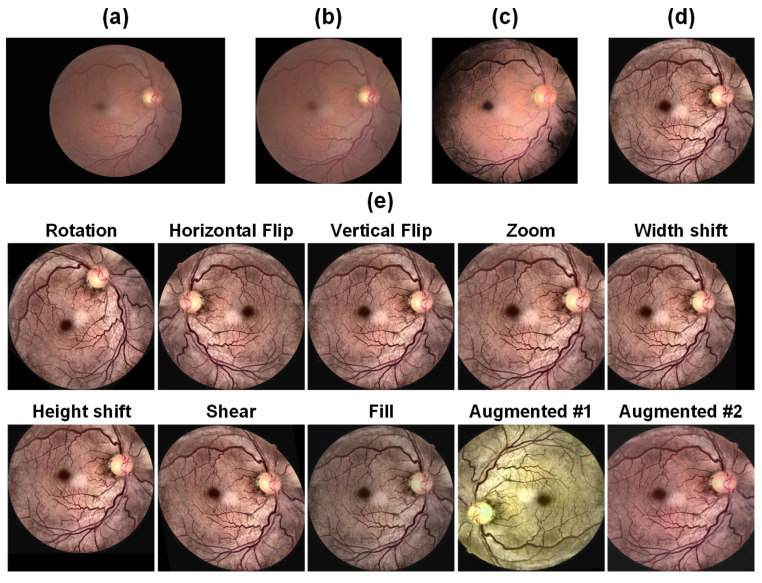
Visualization results of data processing. Firstly, redundant borders were removed (**a**) to ensure a clean and focused representation. Next, Contrast Limited Adaptive Histogram Equalization (CLAHE) was applied to enhance image quality and improve the visibility of important features (**b**). Subsequently, a Gaussian smoothing filter was employed to reduce noise and refine the details further (**c**). Finally, data augmentation techniques were utilized to generate additional training samples (**d**). (**e**) shows examples of eight different data augmentation transformations. Additionally, the last two images in the lower right corner demonstrate a sample that incorporates all data enhancement methods, with their application determined by random probability.

When acquiring fundus images, various factors, such as pixel count, exposure, and contrast, can differ due to variations in the environment, equipment, and human involvement. Some images may suffer from issues like severe overexposure, underexposure, lens contamination, and out-of-focus jitter. These problems significantly impact the efficacy of the classification model. The quality of data preprocessing plays a vital role in the learning capability of the model and ultimately determines its classification performance. Therefore, enhancing image quality stands as a crucial step in determining the effectiveness of the classification model. In the preprocessing of fundus images, the first essential step involves manually filtering out low-quality images. These images lack comprehensive information regarding retinal structure and fail to provide useful pathological features for model learning. We have randomly selected a fundus image in Figure 7 to visualize the changes after the data processing process.

#### 3.2.2. Removing Redundant Borders

Black redundant borders exist in the fundus image. This region does not contribute effective feature information and does not significantly aid in model parameter training during the training process. To address this, a specific approach is implemented. The image is traversed cyclically, identifying the first valid pixel point in all four directions that is not part of the background region. Subsequently, the image is cropped based on these determined points.

#### 3.2.3. Contrast Limited Adaptive Histogram Equalization (CLAHE)

In fundus images, the concave structure of the retina results in a darker edge region compared to the central region, leading to a significant contrast difference from the center to the edge. To address this issue, it is necessary to enhance the contrast of fundus images prior to model training. In our study, we utilized the CLAHE method, which effectively adjusts contrast by constraining noise amplification in contrast adaptive histogram equalization [15]. This method involves cropping the histogram using a predefined clipping threshold and then repeatedly averaging the distribution across individual gray values until the frequencies associated with all gray values fall below the threshold [15]. For our experiment, we applied the CLAHE algorithm for contrast enhancement preprocessing of diabetic retinal fundus images. The clipping threshold parameter, clipLimit, was set to seven, and the tileGridSize parameter was set to six.

#### 3.2.4. Smoothing Gaussian Filtering

To mitigate the impact of uneven brightness conditions, we employ Gaussian filtering to equalize the brightness and contrast of the image. This aims to minimize discrepancies arising from varying exposures and contrasts, allowing for the capture of the maximum amount of detailed features in the affected areas of the fundus image. The smoothed Gaussian filtering algorithm entails calculation of the results through the weighted average of each pixel value and the surrounding pixels. This specific weighted average calculation method can be expressed as follows:

(16) $${\tilde{x}}_{i}(\sigma )=\kappa (x_{i}-G(x_{i};\sigma )\cdot x_{i})+\delta$$

where $G(x_{i};\sigma )$ denotes the Gaussian smoothing function and *σ* represents the standard deviation, which is used to determine the distribution of the background pixel values in the images. The contrast of the image is improved by optimizing the distribution of pixel values, *δ* represents the intensity, and the pixel values are distributed between 0 and 255. In this experiment, we set the parameters as follows: *α* as 4, *σ* as r/30, and *β* as 128.

#### 3.2.5. Data Augmentation

The primary challenge in detecting EALR lies in the abundance of retinal vessels and the indistinct differences they present. Consequently, a substantial amount of image information must be provided to the neural network to extract more specific and comprehensive lesion features. Although the dataset employed contains a significant volume of fundus image data, inconsistencies exist in the distribution of images across different categories, with the normal category containing a disproportionately large number of fundus images. This disparity may cause the deep learning network to predominantly learn features from that particular category during training, subsequently affecting subsequent classification or detection tasks. Hence, data augmentation is necessary to equalize the number of images in each category, thereby positively influencing the training of subsequent models. Given the abundance of images in the normal category, we solely perform data augmentation on the abnormal category to enhance the robustness of the model. For our experiments, we primarily employ random rotation, horizontal flip, vertical flip, proportional scaling, horizontal shift, vertical shift, shear transformation, and fill processes to augment the sample size and ultimately achieve balanced data distribution across categories.

### 3.3. Training and Validation

To conduct a comprehensive evaluation of MIL-CT and its competing models, minimize the risk of overfitting, and utilize the available EALR dataset effectively, we adopt a five-fold cross-validation approach. This methodology enables a thorough assessment by averaging the performance metrics obtained from five independent runs. All experiments were conducted using the PyTorch framework (version 1.8.1) on an NVIDIA RTX 3090 GPU.

For training the models, we employed a total of 200 epochs. The initial learning rate was set to 1 × 10^−3^, with the Adam optimizer employed and a weight decay of 5 × 10^−4^. A batch size of 32 was used, along with a momentum of 0.9. Further details regarding the optimization process and additional hyperparameters will be discussed in the ablation experiments section.

### 3.4. Evaluation Criteria

We employed a comprehensive range of quantitative metrics to evaluate the performance of the MIL-CT model in detecting EALR. These metrics include accuracy (ACC), precision (PRE), sensitivity (SEN), and specificity (SPE). ACC represents the proportion of correctly classified samples as either EALR or normal, while PRE measures the proportion of true-positive samples among those identified as EALR. SEN reflects the proportion of accurately predicted EALR samples, while SPE indicates the proportion of correctly identified normal samples.

To further assess the balance of the model between SEN and SPE, we utilized visualizations such as the receiver operating characteristic (ROC) curve and its area under the curve (AUC). Additionally, we calculated the F1 score, which is the average of PRE and SEN. A high F1 score close to 1 indicates strong EALR detection performance in terms of ACC.

Furthermore, we employed gradient-weighted class activation mapping (Grad-CAM) [16] to visualize the regions of interest in fundus images, thus facilitating interpretable analysis. Finally, we assessed the parametric number and computational efficiency of the model using the number of parameters (Params) and FLOPs.

## 4. Results

### 4.1. Quantitative Analysis

Table 2 presents the quantitative evaluation results of the proposed model variants in a five-fold cross-validation, demonstrating the effectiveness of integrating CT with MIL-ViT. Initially, we used vanilla ViT as the baseline model and constructed feature extraction backbones for three other models: (1) the CT model, where vanilla ViT serves as a cross-scale feature extractor with patch sizes of 16 and 12 for the F-Branch and C-Branch, respectively; (2) the MIL-ViT model, where vanilla ViT acts as the feature extraction backbone; and (3) the MIL-CT model, which combines the first two models.

The results show that the average ACC, PRE, SEN, SPE, and F1 scores of the CT model are improved by 21.10%, 22.96%, 29.66%, 39.23%, and 22.10%, respectively, compared with the baseline model. By utilizing the CT model for multi-scale feature extraction and employing an MHCA module, the model enhances its ability to perceive global information, improves feature representation, and synthesizes information from different scales, thereby reducing information loss. Consequently, the CT model demonstrates good performance in the EALR detection task.

Likewise, the MIL-ViT model also shows significant improvements relative to the baseline model across various metrics. It achieves average enhancements of 17.70%, 18.90%, 24.56%, 32.20%, and 18.67% for ACC, PRE, SEN, SPE, and F1 scores, respectively. These experimental results validate the advantages of the MIL-ViT model in extracting overall features from fundus images. By introducing the MIL module, the model more accurately understands local details and features, especially the lesions or abnormal regions associated with EALR.

Notably, the MIL-CT model exhibits the most favorable EALR detection results due to the efficient information interaction between the CT and MIL models. Compared to the baseline model, the MIL-CT model achieves mean enhancements of 26.42%, 27.24%, 32.78%, 39.89%, and 27.34% for ACC, PRE, SEN, SPE, and F1 scores, respectively, by combining cross-scale fundus information with multiple instances of bag-level classification results.

**Figure 8:**
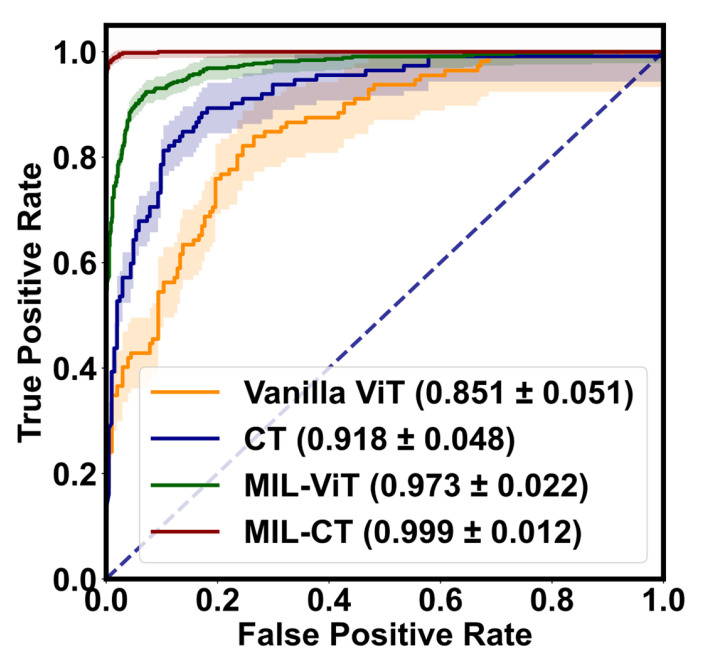
The ROC curves of the variants of the proposed model. The solid line is the mean of the five-fold cross-validation, and the shaded part is the standard deviation.

**Figure 9:**
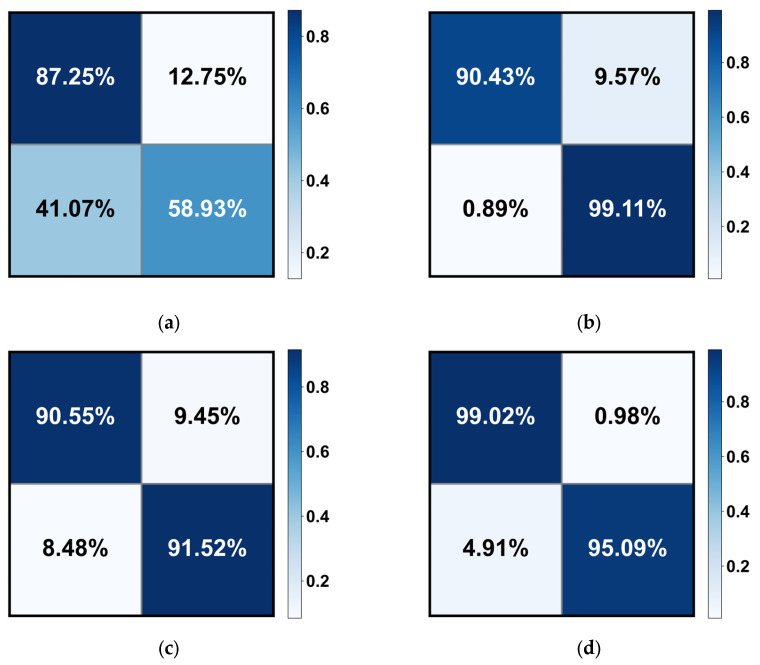
The confusion matrix of the (**a**) vanilla ViT, (**b**) CT, (**c**) MIL-ViT, and (**d**) MIL-CT models for EALR detection. Note that only the results of the median in the five-fold cross-validation are calculated here.

Additionally, we visually depict the performance of the proposed model variants in EALR detection through Figure 8 (ROC curves) and Figure 9 (confusion matrix). These figures highlight the robustness and accuracy of the MIL-CT model.

Therefore, the CT model, the MIL-ViT model, and the MIL-CT model, when combined, demonstrate excellent performance in the study of EALR detection based on fundus images. These models effectively integrate and recognize multiple fine-grained perceptual domains by leveraging the global feature extraction capability of a transformer along with an MIL module. As a result, they offer a scientific approach to the EALR detection problem while significantly improving accuracy and reliability.

### 4.2. Comparison with SOTA Models

Table 3 presents the quantitative evaluation results of our proposed MIL-CT model, comparing its performance with recent parallel works that have achieved advancements in enhancing the efficiency and accuracy of the vanilla ViT model [10]. The results in Table 3 demonstrate that MIL-CT surpasses all other methods with equivalent FLOPs and Params.

Importantly, the F1 score of MIL-CT is notably higher, exhibiting a significant improvement of 23.23% (86.98% vs. 97.62%) compared to the second-ranked MobileViT model [17], despite the increased FLOPs and Params. Additionally, MIL-CT showcases similar computational efficiency and Params to EfficientFormer [18] while outperforming other models in terms of F1 score, with improvements ranging from 16.12% to 84.47%. Therefore, our method consistently outperforms all models except for MobileViT [17] and EfficientFormer [18] in terms of accuracy and parameter count, validating further the efficacy of multi-scale features in transformers. In comparison to other transformer models, MIL-CT achieves superior accuracy with fewer parameters, thereby reducing computational complexity.

In light of the dominant position of computer vision applications and the increasing demand for fundus image analysis, we deployed various CNN architectures, including popular models such as the hand-crafted ResNet [25] and the search-based EfficientNet [26,27], to comprehensively compare their performance with our proposed MIL-CT model. The results of the comparative experiment are presented in Table 4.

First, MIL-CT exhibits superior accuracy, efficiency, and compactness when compared to the ResNet family of models (ResNet [25], ResNeXt [28], SEResNet [29], and ECAResNet [30]). Specifically, MIL-CT outperforms these models in terms of ACC, PRE, SEN, SPE, and F1 score, except for MobileNetv3 [27], which is marginally faster. Notably, even after increasing Params in our CNN model to enhance its fitting ability, MIL-CT remains remarkably competitive. Its improvement ranges are approximately 13.00% to 32.96%, 13.04% to 34.24%, 12.82% to 42.34%, and 15.31% to 54.05%, respectively. Furthermore, our model achieves an F1 score higher than 86.38%, emphasizing its crucial role in enhancing EALR detection, particularly given that most competing models fall below this threshold by 11.51%. We are optimistic that, by incorporating neural architecture search methods [26,27], our approach can bridge the Params gap with EfficientNet [26].

### 4.3. Ablation Studies

#### 4.3.1. Effect of Patch Sizes

MIL-CT utilizes MHCA to establish global correlations among fundus images at various scales, effectively capturing information from multiple perceptual domains of different granularity. This approach significantly enhances the detection of small-sized fundus lesions. To assess the impact of patch size on EALR detection performance, we conducted experiments using (8, 16) and (12, 16) as the patch sizes in the F-Branch and C-Branch, respectively. Based on the results presented in Table 5, we observed that MIL-CT with a patch size of (12, 16) outperforms MIL-CT-1 in EALR detection while requiring fewer patches. This implies that excessively pursuing fine-grained features may result in a significant scale difference between the two branches, thereby affecting the smooth learning of features (with the number of patch tokens in both branches being doubled). Hence, selecting an appropriate patch size is crucial for improving detection accuracy in studies focusing on EALR detection using fundus images.

#### 4.3.2. Effect of the F-Branch

When conducting EALR detection using MIL-CT, a multi-scale approach is employed to capture lesions of varying patch sizes. Larger lesions can be effectively detected by the C-Branch, while smaller lesions require the F-Branch for detection. In order to balance the computational load, we designed the F-Branch to be shallower and narrower compared to the C-Branch. To investigate whether increasing the complexity of the F-Branch would lead to performance improvements, we designed the MIL-CT-2 and MIL-CT-3 models, and their results are presented in Table 5. Despite both models having increased Params and FLOPs, there was no significant improvement in EALR detection performance. In addition, our modeling test results in Appendix B regarding the retention of only the F-Branch and C-Branch are consistent with this conclusion. We believe that there are three potential reasons for this:

1. Information redundancy: The F-Branch is specifically designed to handle smaller scale lesions efficiently. If the width and depth of the fine-grained branch are increased excessively, it may introduce an abundance of parameters, resulting in information redundancy. These redundant parameters can hamper the training process of the model and make it challenging to learn meaningful features.
2. Localization problem: The F-Branch focuses more on the intricate details and local information of the image. However, in EALR detection, smaller scale lesions may lack distinctive global features. Consequently, deepening the fine-grained branch may not provide additional valuable information since it is better suited to address image details rather than global features.
3. Limitations in additional information: Typically, the F-Branch only provides simplistic supplementary information about the fundus image. Due to its narrower width and shallower depth, this branch possesses relatively limited representational power and may only capture a few basic features in the image. Even if the fine-grained branch is deepened, its capacity might still be insufficient to learn more complex and abstract features, thereby failing to enhance the performance of model.

Hence, even with increased width and depth in the F-Branch, there may not be a noticeable enhancement in the performance of the model. Instead, the F-Branch might be better suited to providing complementary features in fundus images, which can then be fused with the more intricate and abstract features captured by the C-Branch, resulting in more accurate EALR detection.

#### 4.3.3. Effect of the Number of CT Encoders

In investigating the detection of EALR in fundus images, the presence of spatial correlation and long-range dependence emerges as a significant feature. To effectively capture the mutual information between different patches, MIL-CT incorporates CT encoders that utilize the MHCA mechanism. These encoders aim to model global spatial relationships and facilitate information transfer across distant regions. In order to evaluate the impact of CT encoders on the performance of MIL-CT, we attempted to stack CT encoders with four, five, and six layers to construct the MIL-CT-4, MIL-CT-5, and MIL-CT-6 models, respectively.

However, experimental results indicated that including more CT encoders did not result in a significant performance improvement compared to the baseline model (MIL-CT model with three layers of CT encoders). The F1 scores of the MIL-CT-4, MIL-CT-5, and MIL-CT-6 models were found to be 0.40–0.76% lower than the baseline model. Additionally, the inclusion of additional CT encoders introduced unnecessary complexity without providing significant benefits. These results suggest that MIL-CT already possesses sufficient capacity to capture features in fundus images, making the incorporation of extra CT encoders redundant.

To address concerns such as overfitting and resource inefficiency, we recommend adjusting the capacity of MIL-CT appropriately when conducting EALR detection, rather than blindly adding more CT encoders. This approach ensures that the model maintains adequate expressive power while avoiding unnecessary complexity.

#### 4.3.4. Sensitivity to MIL

The patch token-based bag-level classifier loss method employs the MIL technique [27] to divide the input image into multiple patches and independently calculate the cross-entropy (CE) loss for each patch. This approach enhances the perception of different regions by classifying each patch individually, thereby better capturing local features and detailed information. In this study, we aim to apply patch token-based bag-level classifier loss to the fundus image-based EALR detection problem.

To adjust the weighting relationship between patch token-based bag-level classifier loss and classification token loss, we introduced the hyperparameter *η*, which varies between 0.2 and 1.0. This was chosen in order to achieve a balance between the importance of the two losses. Through our ablation experiments, we investigated the impact of different values of *η* on EALR detection performance and conducted sensitivity analysis. The experimental results are presented in Table 6. We observe that when the weight of the classification token loss is too low (close to 0), the model prioritizes the patch token-based bag-level classifier loss, neglecting the importance of the balanced classification token loss. The classification token loss serves to model global spatial relationships and facilitate information transfer through an MHCA mechanism, enabling a better representation of interrelationships between lesions. Consequently, if the weight of the classification token loss is too small, the model fails to fully leverage this global information transfer mechanism, leading to a decline in performance.

However, as the weight of the classification token loss increases, the model strikes a balance between both losses, allowing for a synergistic combination of global information and local features. With higher weights assigned to the classification token loss, the model places greater emphasis on modeling global information, resulting in gradual improvements in EALR detection performance. When the weight attains its optimal value (0.6), the model achieves peak performance while maintaining a proper equilibrium between global information and local features. Nevertheless, exceeding the optimal weight value for the classification token loss (greater than 0.6) causes a progressive increase in the relative importance of the patch token-based bag-level classifier loss. This overemphasis on local features may lead to neglect in terms of modeling global relationships, resulting in a gradual deterioration in performance. Consequently, an excessive weight for the classification token loss can negatively impact overall model performance.

In conclusion, the patch token-based bag-level classifier loss method effectively captures local features and detailed information in fundus images, while the classification token loss method models global spatial relationships. By appropriately adjusting the weights of the classification token loss, the importance of global information and local features can be balanced, thereby improving EALR detection performance.

#### 4.3.5. Combination of Pre-Training

After conducting an experimental evaluation, we explored the impact of different pre-training strategies on the performance of lesion detection in fundus images. These strategies comprised the baseline model (without the use of pre-trained weights), the contrast model (utilizing weights pre-trained on ImageNet [35]), and our proposed model (employing weights pre-trained on the large fundus image Kaggle dataset [14]).

According to the findings presented in Table 7, it is evident that both the contrast models and the model using ImageNet pre-trained weights achieved lower F1 scores in EALR detection compared to our proposed model, with reductions of 2.68% and 1.15%, respectively.

In the baseline model, we opted not to implement any pre-trained weights. Specifically, we initialized the weights from scratch and trained them using the EALR dataset. However, this approach may not effectively capture the relevant features associated with lesions due to the limited nature of the fundus image.

Conversely, utilizing weights pre-trained on ImageNet can provide some generic visual features for comparison models, which prove effective for common object classification tasks. Nevertheless, since the features of EALR lesions differ from those involved in the object classification tasks of ImageNet, these pre-trained weights may not sufficiently capture the specific features attributed to EALR in fundus images.

On the other hand, the utilization of weights pre-trained on the large fundus image Kaggle dataset proves more suitable for the EALR detection task compared to the previous two cases. These weights are acquired through pre-training on a domain similar to the fundus image dataset, enabling them to better capture the features associated with EALR lesions. This pre-training strategy enables the model to learn a representation more tailored to the EALR detection task, resulting in improved generalization on new fundus image data and ultimately yielding better performance.

## 5. Discussion

### 5.1. Interpretability Analysis

In this study, we utilized Grad-CAM to visualize the attention regions of various models in the EALR detection task. Grad-CAM calculates the importance weights of each pixel by multiplying the output of the model with the gradient between the feature maps using backpropagation and global average pooling operations. These weights generate color maps that highlight the model’s regions of interest in the fundus image.

**Figure 10:**
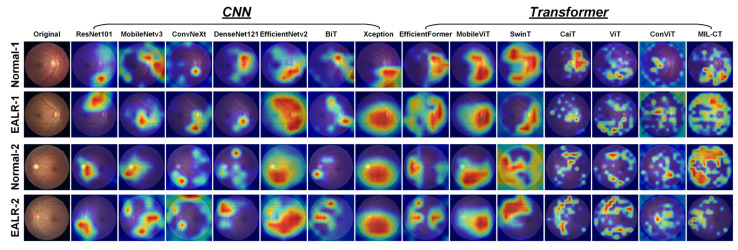
The attention regions of CNN-based and transformer-based models in EALR detection.

As shown in Figure 10, analyzing the Grad-CAM attention heat maps of different models reveals that CNN-based architectures (ResNet101 [25], MobileNetv3 [27], ConvNeXt [33], DenseNet121 [34], EfficientNetv2 [26], BiT [31], and Xception71 [32]) tend to focus on retinal vascular coverage regions and irrelevant background areas. Due to their local receptive fields and the nature of convolutional layers, CNN models are inclined to learn from local information and specific details, potentially ignoring global lesion features in EALR detection tasks. Conversely, transformer-based models (EfficientFormer [18], MobileViT [17], SwinT [23], CaiT [20], vanilla ViT [10], ConViT [24]) primarily concentrate on continuous regions, thus capturing global features. However, these models often struggle to accurately identify important regions associated with lesions, possibly due to distractions present in continuous areas.

Based on these observations, we propose the MIL-CT model, which overcomes the limitations of focusing solely on local or global features. MIL-CT accurately focuses on the morphological changes in retinal vessels, which is critical for EALR detection. This is achieved through a transformer encoder with an MHCA mechanism, enabling the model to capture global spatial relationships and transfer information across different perceptual domains. By doing so, MIL-CT effectively captures the interrelationships between vessels exhibiting different morphologies. Furthermore, MIL-CT incorporates MIL and introduces a novel bag-level classifier. This approach maximizes the potential of the feature representations extracted from individual patches, which are often neglected by transformer-based models. Consequently, MIL-CT enhances the detection of morphological features associated with EALR.

Considering the aforementioned observations and experimental results, we conclude that MIL-CT outperforms traditional CNN and transformer models in the EALR detection task. It achieves this by capturing cross-scale features and patch token features through an MHCA mechanism and an MIL framework based on MIL. Additionally, MIL-CT prioritizes the morphological features of blood vessels associated with EALR. These advantages establish MIL-CT as an effective model for EALR detection.

### 5.2. Comparison with Competitive Works

To the best of our knowledge, this is the first study on EALR detection. It analyzes vessel features in fundus images to assess ocular health by extracting information about vessel structure, density, and distribution through the analysis of light-reflected vessel features. However, numerous studies have proposed competitive deep learning models for the detection of diabetic retinopathy (DR). Therefore, we applied these methods to the EALR dataset to evaluate their suitability for EALR detection. The following provides a brief description of these SOTA models:

1. Scratched-CNN-1 [36]: Zago et al. employed a pre-trained VGG16 model and a custom CNN for DR detection. The custom CNN consists of five convolutional layers, five maximum pooling layers, and one FC layer. This model achieved the best SEN value of 0.94 with an AUC of 0.912.
2. WP-CNN [37]: Liu et al. developed a weighted path CNN (WP-CNN) to detect DR images requiring referral. The WP-CNN incorporates multiple convolutional layers with different kernel sizes in different weighted paths, which are then merged through averaging. Results show that the WP-CNN has higher SEN compared to pre-trained ResNet, SeNet, and DenseNet structures, with higher ACC of 94.23%.
3. CNN-Adaboost [38]: Jiang et al. integrated three pre-trained CNN models, Inceptionv3, Inception-ResNetv2, and ResNet152, to classify the DR dataset into DR requiring referral and DR not requiring referral. Training of the CNN utilized the Adam optimizer to update weights and was integrated using the Adaboost algorithm. This method achieved an ACC of 88.21% with an AUC of 0.946.
4. CNN-Ensemble-1 [39]: Qummar et al. trained five integrated CNN models with deep convolution (ResNet50, Inceptionv3, Exception, DenseNet121, DenseNet169) using the publicly available Kaggle retinal image dataset to encode rich features and improve the classification of different stages of DR.
5. SDL [40]: Shankar et al. proposed a deep learning-based model for automatic detection and classification of fundus DR images. They preprocessed the fundus image dataset by removing noise, performing histogram segmentation, and extracting regions of interest. Subsequently, they employed a collaborative deep learning (SDL) model to hierarchically classify the DR fundus images.
6. CNN-Ensemble-2 [41]: Bellemo et al. utilized an integrated CNN model consisting of two CNNs (an adaptive VGGNet structure and residual neural network structure) to classify fundus images. The AUC of this integrated CNN was 0.723, while the expert classification result achieved an AUC of 0.741.
7. GWO-DNN [42]: Gadekallu et al. used a deep neural network model based on principal component analysis to classify features extracted from the DR dataset using the grey wolf optimization (GWO) algorithm. They normalized the dataset using the standard scaler normalization method and employed principal component analysis for dimensionality reduction. They selected the best hyperparameters through GWO and finally trained the dataset using a deep neural network (DNN) model. Results showed that the model outperformed traditional machine learning algorithms.
8. ResNet-Attention [43]: Li et al. employed ResNet50 and four attention modules to classify the fundus dataset in terms of requiring referral or not. The best results achieved by this model included 92% SEN, 96.3% AUC, and 92.6% ACC.
9. Scratched-CNN-2 [44]: Mobeen et al. used their self-created CNN architecture along with pre-trained models (including AlexNet, VGG-16, and SqueezeNet) to detect DR classes in the fundus dataset. Their CNN architecture achieved the highest ACC of 98.15%, specificity (SPE) of 97.87%, and SEN of 98.94%.
10. M-R-FCN [45]: Wang et al. enhanced R-FCN by incorporating a feature pyramid network and five region suggestion networks, achieving an SEN of 92.59% for DR detection on the fundus dataset.

To assess the adaptability of these competitive methods for EALR detection, we replicated these models and evaluated them using the EALR dataset. The results are summarized in Table 8. As depicted in the results, our MIL-CT method outperforms all SOTA techniques in terms of EALR detection performance. It improves ACC, PRE, SEN, SPE, and F1 score by 4.93–32.85%, 4.87–31.36%, 6.26–34.12%, 7.63–35.41%, and 5.02–32.33%, respectively, bringing them to 97.62%, 97.63%, 97.05%, 96.48%, and 97.62%, respectively. Consequently, we argue that DR and EALR are distinct types of ocular lesions, and their characteristics and physiological processes may differ. Therefore, the effectiveness of transfer learning may be limited. Additionally, the model may have learned some features from the DR study that are not relevant to EALR, resulting in decreased performance in EALR detection. Furthermore, this approach may encounter challenges related to model uncertainty and interpretation. Thus, our approach has the potential to assist physicians in accurately diagnosing and treating EALR, thereby reducing patient suffering and financial burden.

### 5.3. Limitation and Future Work

Although our MIL-CT method has presented promising results in detecting EALR using fundus images, there is still room for improvement. Firstly, we have only tested a limited number of models and have not conducted a comprehensive evaluation of all possible models. Therefore, it is necessary to expand our testing to include a wider range of vision models with different sizes and architectures. Additionally, exploring knowledge sharing and transfer among different models can further enhance the performance and generalization capabilities of these models. Finally, while most studies have utilized data augmentation methods to address the imbalance in fundus images, our preliminary experiments in Appendix A suggest that bootstrap-based oversampling methods seem to improve the generalization of the model. However, this conclusion still requires validation on a broader dataset.

For future work, we plan to validate the effectiveness of the MIL-CT method using a larger sample size. Furthermore, we aim to develop more accurate models specifically designed for EALR detection. Additionally, we will explore other architectural search strategies and knowledge distillation methods to improve performance and reduce model complexity. By incorporating these improvements, we anticipate that the effectiveness of the MIL-CT method in EALR detection tasks will be enhanced, providing reliable tools for the diagnosis and treatment of chronic diseases related to retinal disorders.

## 6. Conclusions

In this study, we propose a novel CT-based MIL method called MIL-CT for detecting EALR. MIL-CT leverages the CT backbone to extract retinal features in the multi-granularity perceptual domain. The method incorporates an MHCA fusion module to enhance global perceptual capability, feature representation, and information integration across different scales. This approach effectively reduces information loss and improves the performance of the EALR detection task. Furthermore, our proposed MIL module enables the model to better comprehend local details and features in fundus images. It accurately classifies the features of patch tokens. By combining classification tokens and multi-instance classification results, the method considers both global and local information, resulting in improved accuracy in EALR detection.

Through ablation experiments, comparison experiments, and interpretability analysis experiments conducted on fundus image datasets, we demonstrated that EALR effectively reduces generalization errors while efficiently attending to retinal vessel details. Additionally, our method achieves SOTA performance, outperforming all proposed models in EALR detection. These results highlight the potential of the MIL-CT method in significantly enhancing the diagnostic accuracy of fundus images, particularly in EALR detection. This contribution is valuable in terms of the early screening of cardiovascular diseases such as hypertension and atherosclerosis.

## Figures and Tables

**Table 1:** Data distribution of Kaggle and EALR datasets.

Source	Category	Stage	Training Set	Testing Set	Total	Proportion
Kaggle [14]	0	Normal	23,814	39,553	63,367	72.61%
	1	Mild NPDR	2444	3762	6206	7.11%
	2	Moderate NPDR	5294	7861	13,155	15.07%
	3	Severe NPDR	873	1214	2087	2.39%
	4	PDR	708	1206	1914	2.21%
EALR	0	Normal	815	204	1019	64.53%
	1	EALR	448	112	560	35.47%

**Table 2:** Quantitative evaluation results of the variants of the proposed model.

Model Variant	Type	ACC (%)	PRE (%)	SEN (%)	SPE (%)	F1 (%)
Vanilla ViT [10]	Normal	77.22	79.46	87.25	58.93	83.18
	EALR	77.22	71.74	58.93	87.25	64.71
	Average	77.22	76.73	73.09	68.97	76.66
CT	Normal	93.51	99.46	90.43	99.11	94.73
	EALR	93.51	85.06	99.11	90.43	91.55
	Average	93.51	94.35	94.77	96.03	93.60
MIL-ViT	Normal	90.89	95.10	90.55	91.52	92.77
	EALR	90.89	84.19	91.52	90.55	87.70
	Average	90.89	91.23	91.04	91.18	90.97
MIL-CT	Normal	97.62	97.35	99.02	95.09	98.18
	EALR	97.62	98.16	95.09	99.02	96.60
	Average	97.62	97.63	97.05	96.48	97.62

**Table 3:** Quantitative comparison results with SOTA transformer models.

Model	ACC (%)	PRE (%)	SEN (%)	SPE (%)	F1 (%)	Params (M)	FLOPs (G)
Vanilla ViT [10]	77.22	76.73	73.09	68.97	76.66	102.44	16.88
MobileViT [17]	87.03	86.96	85.52	84.02	86.98	5.57	1.42
EfficientFormer [18]	84.18	84.04	81.35	80.04	84.07	31.89	3.94
CaiT [19]	74.05	73.61	76.20	67.23	73.75	46.82	8.63
XCiT [20]	71.52	70.46	73.17	59.40	70.07	47.64	8.92
BEiT [21]	75.32	75.22	74.28	70.34	75.27	81.18	12.70
VOLO [22]	75.32	74.77	75.55	64.30	74.16	58.58	13.61
SwinT [23]	61.71	52.83	65.32	37.10	52.92	109.07	15.19
ConViT [24]	76.58	76.49	77.81	71.84	76.53	86.39	16.81
MIL-CT (our)	97.62	97.63	97.05	96.48	97.62	32.35	6.51

**Table 4:** Quantitative comparison results with SOTA CNN models.

Model	ACC (%)	PRE (%)	SEN (%)	SPE (%)	F1 (%)	Params (M)	FLOPs (G)
ResNet101 [25]	81.65	81.41	81.65	74.62	81.23	44.55	7.87
ResNeXt101 [28]	73.42	72.73	69.14	64.87	72.82	88.79	16.54
SEResNet101 [29]	73.73	72.96	68.18	62.63	72.55	49.33	7.64
ECAResNet101 [30]	73.73	72.95	68.38	63.03	72.65	44.57	8.11
MobileNetv3 [27]	84.49	84.46	84.57	81.42	84.48	4.18	0.22
BiT [31]	76.90	76.56	77.12	70.81	76.67	44.76	0.04
Xception71 [32]	83.86	83.67	84.13	78.66	83.66	42.33	9.88
EfficientNetv2 [26]	86.39	86.37	86.02	83.67	86.38	19.22	1.50
ConvNeXt [33]	74.68	74.41	73.17	68.79	74.52	50.18	8.68
DenseNet121 [34]	85.44	85.58	84.25	83.55	85.50	7.90	2.83
MIL-CT (our)	97.62	97.63	97.05	96.48	97.62	32.35	6.51

**Table 5:** Ablation experiment with different architecture parameters. The underlined values indicate changes from MIL-CT.

Model	Patch Size		Dimension		F	C	T	ACC (%)	F1 (%)	Params (M)	FLOPs (G)
	F-Branch	C-Branch	F-Branch	C-Branch							
MIL-CT-1	8	16	96	192	1	4	3	97.35	97.13	32.35	7.53
MIL-CT-2	12	16	192	192	1	4	3	96.75	96.40	38.91	8.60
MIL-CT-3	12	16	96	192	2	4	3	97.40	97.31	33.82	7.25
MIL-CT-4	12	16	96	192	1	4	4	97.53	97.23	33.25	6.51
MIL-CT-5	12	16	96	192	1	4	5	97.28	96.95	34.79	6.51
MIL-CT-6	12	16	96	192	1	4	6	97.05	96.88	35.17	6.51
MIL-CT (our)	12	16	96	192	1	4	3	97.62	97.62	32.35	6.51

**Table 6:** Experimental results of sensitivity analysis for the loss hyperparameter of MIL.

*η*	ACC (%)	PRE (%)	SEN (%)	SPE (%)	F1 (%)
0.2	95.17	95.17	94.70	94.23	95.17
0.4	96.36	96.35	95.82	95.28	96.35
0.5	97.62	97.63	97.05	96.48	97.62
0.6	97.74	97.89	97.36	96.18	97.81
0.8	94.46	94.49	94.15	93.84	94.47
1.0	93.51	94.35	94.77	96.03	93.60

**Table 7:** Effect of different pre-training experiments on ELAR detection performance.

Pre-Trained Dataset	ACC (%)	PRE (%)	SEN (%)	SPE (%)	F1 (%)
Without Pre-training	95.01	95.00	94.38	93.74	95.00
ImageNet [35]	96.52	96.52	95.79	95.07	96.50
Kaggle [14]	97.62	97.63	97.05	96.48	97.62

**Table 8:** Experimental results on competitive models.

Work	Model	ACC (%)	PRE (%)	SEN (%)	SPE (%)	F1 (%)
Zago et al. [36]	Scratched-CNN-1	75.85	76.22	74.30	72.75	76.00
Liu et al. [37]	WP-CNN	74.98	75.44	73.48	71.97	75.16
Jiang et al. [38]	CNN-Adaboost	78.70	78.76	76.86	75.02	78.73
Qummar et al. [39]	CNN-Ensemble-1	73.48	74.32	72.36	71.25	73.77
Shankar et al. [40]	SDL	81.31	81.69	80.40	79.48	81.45
Bellemo et al. [41]	CNN-Ensemble-2	79.33	79.51	77.81	76.28	79.41
Gadekallu et al. [42]	GWO-DNN	87.09	87.04	85.68	84.26	87.06
Li et al. [43]	ResNet-Attention	91.92	91.95	90.17	88.42	91.83
Mobeen et al. [44]	Scratched-CNN-2	93.03	93.10	91.33	89.64	92.95
Wang et al. [45]	M-R-FCN	85.83	85.88	84.70	83.57	85.85
Our proposed model	MIL-CT	97.62	97.63	97.05	96.48	97.62

## Data Availability

The data presented in this study are available on request from the corresponding author. The data are not publicly available due to the privacy of patients being protected.

## Appendix A

The EALR dataset suffers from a severe data imbalance issue, and most studies address this problem in fundus images through data augmentation methods. However, these methods may potentially reduce the model’s generalization ability. In particular, bootstrap-based oversampling methods do not impose constraints on the probability distributions of the original samples. They aim to obtain probabilities that closely resemble the true distribution of the data, thus appearing to enhance the model’s generalization ability. Hence, we investigated the impact of bootstrap methods on EALR detection performance. To this end, we implemented the bootstrap method based on the maximum entropy principle proposed by Lei et al. [46]. Firstly, we obtained the probability distribution of fundus image samples using the bootstrap method and optimized it using the maximum entropy principle. Secondly, we applied a probability enhancement algorithm based on the distribution of a few classes of samples. This ensured consistency in the probability densities of EALR samples before and after the dataset was balanced, thereby improving the effectiveness of MIL-CT classification. The experimental results presented in Table A1 demonstrate a slight improvement in ACC, PRE, SEN, and F1 scores for the bootstrap-based method compared to the image augmentation-based method. These findings suggest that the bootstrap-based oversampling method enhances the model’s generalization ability. Nevertheless, further validation of this conclusion requires a broader dataset.

**Table A1:** Effect of different algorithms for handling data imbalance experiments on ELAR detection performance.

Algorithm	ACC (%)	PRE (%)	SEN (%)	SPE (%)	F1 (%)
Bootstrap	97.82	97.70	96.79	96.83	97.71
Image Augmentation	97.62	97.63	97.05	96.48	97.62

## Appendix B

Attentional information fusion plays a critical role in acquiring EALR-related information in retinal images. The experimental results presented in Table A2 demonstrate that the proposed MIL-CT approach outperforms the F-Branch-only and C-Branch-only approaches, with an improvement of 0.64% and 1.93%, respectively, in terms of the F1 score. We assert that the cross-scale attention fusion module enhances the model’s global perception and feature characterization ability by integrating information from different scales while minimizing information loss. This enables more effective detection and classification of EALR-related features. For instance, subtle variations in arterial light reflection characteristics across various scales can be captured and utilized efficiently for accurate detection.

**Table A2:** Effect of model branching on ELAR detection performance for different granularity sensory domains.

Algorithm	ACC (%)	PRE (%)	SEN (%)	SPE (%)	F1 (%)
F-Branch Only	97.05	96.93	96.82	96.28	97.00
C-Branch Only	96.29	96.35	95.93	95.28	95.77
CT	97.62	97.63	97.05	96.48	97.62
